# Detecting methanol in hand sanitizers

**DOI:** 10.1016/j.isci.2021.102050

**Published:** 2021-01-12

**Authors:** Andreas T. Güntner, Leandro Magro, Jan van den Broek, Sotiris E. Pratsinis

**Affiliations:** 1 Particle Technology Laboratory, Department of Mechanical and Process Engineering, ETH Zurich, CH-8092 Zurich, Switzerland

**Keywords:** Chemistry, Analytical Chemistry, Chemical Composition Analysis

## Abstract

The coronavirus disease 2019 (COVID-19) pandemic has increased dramatically the demand for hand sanitizers. A major concern is methanol adulteration that caused more than 700 fatalities in Iran and U.S.A. (since February 2020). In response, the U.S. Food and Drug Administration has restricted the methanol content in sanitizers to 0.063 vol% and blacklisted 212 products (as of November 20, 2020). Here, we present a low-cost, handheld, and smartphone-assisted device that detects methanol selectively in sanitizers between 0.01 and 100 vol% within two minutes. It features a nanoporous polymer column that separates methanol selectively from confounders by adsorption. A chemoresistive gas sensor detects the methanol. When tested on commercial sanitizers (total 76 samples), methanol was quantified in excellent (R^2^ = 0.99) agreement to “gold standard” gas chromatography. Importantly, methanol quantification was hardly interfered by sanitizer composition and viscosity. This device meets an urgent need for on-site methanol screening by authorities, health professionals, and even laymen.

## Highlights

• Inexpensive and handheld methanol sensor for hand sanitizer analysis
• Detects 0.01–100 vol% methanol within two minutes
• Robust to different sanitizer compositions and viscosities
• Smartphone-assisted and readily applicable by laymen

## Graphical abstract

**Figure:**
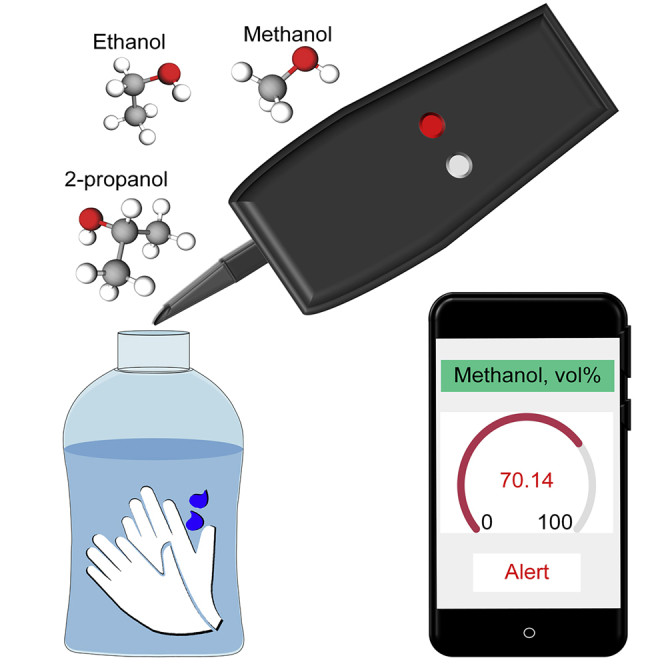

## Introduction

The global health emergency due to the infectious severe acute respiratory syndrome coronavirus 2 (SARS-CoV-2) causing COVID-19 (Wu et al., 2020) has rapidly increased the need for personal protective equipment (e.g. face masks, ventilators, or sanitizers), that led temporarily to acute shortages in supply compromising health-care workforce safety (Ranney et al., 2020). In case of hand sanitizers, global production has grown involving also small businesses (e.g. distilleries) and universities (Dicken et al., 2020) that produce and distribute hand sanitizers often locally at small scale. In fact, the hand sanitizer market is expected to be 4.5 and 2.2 Billion USD in the Asia Pacific region (Renub Research, 2020a) and U.S (Renub Research, 2020b), respectively, by 2026. Public awareness about safety issues in hand sanitizers has emerged since the FDA placed a warning for 212 products (by November 20, 2020) (U.S. Food and Drug Administration, 2020a) that contained up to 81 vol% of toxic methanol, drastically exceeding recommended (U.S. Food and Drug Administration, 2020b) limits (0.063 vol%). Similar hand sanitizer concerns have been published by the Canadian government (Government of Canada, 2020). The ingestion of methanol-contaminated sanitizers led already to more than 700 fatalities in Iran (Wambua-Soi, 2020) and the U.S.A. (Fazio, 2020) since February 2020.

Commercial hand sanitizers should contain only ethanol or 2-propanol for antisepsis, according to the World Health Organization (WHO) (World Health Organization, 2010). For instance, after 30 s, the viral infectivity of SARS-CoV was reduced by more than 4 or 3 orders of magnitude with 80 vol% ethanol or 70 vol% 2-propanol, respectively (Kampf et al., 2020). Other substances like glycerol (humectant), hydrogen peroxide (against bacterial spores), odorants and colorants may be contained as well (World Health Organization, 2010). Methanol is colorless and hardly distinguishable by odor from other alcohols like ethanol, so it cannot be recognized easily by human olfaction or vision. Its toxicity is primarily related to its metabolic products formaldehyde and formic acid (Barceloux et al., 2002) that can cause permanent neurologic dysfunctions, ocular morbidity up to blindness or even death (Kraut and Mullins, 2018). Therefore, low-cost and portable methanol detectors are needed to assist distributors, local authorities and even consumers to check product safety. Analytically challenging for such detectors are the required selectivity over other hand sanitizer ingredients, the large methanol detection range (at least 0.063–81 vol%), fast response times and, ideally, repeated usability.

Gas or liquid chromatography are most established for methanol detection in complex mixtures, but these are bulky, expensive instruments that require trained personnel (Kraut and Kurtz, 2008), usually available only in specialized laboratories and unsuitable for on-site analyses (Kraut and Mullins, 2018). Also optical infrared detectors suffer from similar drawbacks, for instance, the Spectrum Two FT-IR Spectrometer (PerkinElmer) and DX4000/DX4015 (Gasmet Technologies) weigh 13 and 15 kg, respectively, and such instruments are rather expensive (tens of thousands of USD (U.S. Department of Defense, 2020)). Cheaper, more compact, and less power consuming (Güntner et al., 2020) are chemical gas sensors (e.g. Pt-loaded tungsten nitride (Meng et al., 2020), polymer-coated Si bridges (Guo et al., 2011), electrochemical cells (Ou et al., 2019), or nanoporous Al_2_O_3_-coated carbon nanotubes (Zhao et al., 2012)) that detect methanol from the headspace of liquids. However, most are interfered by ethanol that is usually present at high content (Table 1), and none has been tested on hand sanitizers (Table 2). Finally, a colorimetric assay (Alert for Methanol, Neogen Corp., ca. 20 USD per test) is available for alcoholic beverage analysis, which indicates if methanol is below or above 0.35 vol% but is insufficient to check FDA adherence. Also, it is single-use, requires cooling during storage (2–8 °C) and might be interfered particularly by colorants (sample #6 contains patent blue V, Table 1) but also other hand sanitizer ingredients (e.g. 2-propanol, glycerol, odorants) and may fail on gel-like hand sanitizers (such as sample #7).

**Table 1:** Hand sanitizer compositions

Brand	Sample	Composition (vol%)
B. Braun Medical	#1	Ethanol (85), glycerol (0.7), butanone (<3)
∗WHO	#2	Ethanol (72), glycerol (1.45), hydrogen peroxide (0.125), rest water
Martec Desinfektion	#3	Ethanol (82)
Lactipar Desin Händedesinfektion	#4	Ethanol (>80), butanone (<5.3)
Conviva Händedesinfektionsmittel	#5	Alcohol denat. (81), water, glycerol, panthenol, cyclopentasiloxane, cyclohexasiloxane, isotrideceth-8, 2-propanol, didecyldimethylammoniumchloride (0.05)
Sterillium	#6	2-propanol (49), 1-propanol (32) mecetroniumetilsulfat (0.2), glycerol, tertradecanol, odorants, patent blue V, water
Martec Hand-Desinfektion Gel	#7 (gel)	Ethanol (71.5), aloe vera essence

Commercial hand sanitizers and their composition, as indicated by supplier. Contents by volume are indicated in brackets, if available.

∗Mixed according to WHO hand rub formulation (World Health Organization, 2010) but with fruit spirit-derived ethanol.

**Table 2:** Performance of compact methanol detectors

Type	Reference	LOQ^a^ (vol%)	Analysis time (s)	Methanol selectivity^b^					Reusable^c^	Stability^d^ (days)	Validated with hand sanitizers	Price (USD)
				Ethanol	1-propanol	2-propanol	Butanone	Glycerol				
Chemoresistive	Guo et al., (2011)	0.02 (g)		0.5					✓			
	Zhao et al., (2012)	8∙10^−7^ (g)		<2					✓			
	This work	0.01 (L) 10^−4^ (g)^g^	≤90	∞	∞	∞	∞	∞	✓	107^i^	✓	
EC^e^	Ou et al., (2019)	0.15 (L)		260					✓			
	Meng et al., (2020)	2∙10^−4^ (g)	60	~1					✓	15		
Optical	DX4015 (Gasmet Technol.)	3∙10^−4^ (g)	<120						✓			>10′000
	Spectrum Two FT-IR Spectrometer (PerkinElmer)	0.03 (L)	30						✓		✓	
	Huang et al., (2018)	4 (L)	<2	0.7^h^					✓			
CM^f^	Alert for Methanol (Neogen)	0.35 (L)	600						single use			20 (per analysis)

a lowest gas- (g) or liquid- (L) phase concentration measured.

b highest ratio of response methanol vs. response confounder.

c repeated use of same detector/reagent.

d stability during repeated measurements without significant performance loss.

e electrochemical.

f colorimetric.

g data from van den Broek et al. (2019).

h authors suggest ethanol vs. methanol discrimination through different sensor recovery times.

i data from Abegg et al. (2020).

Here, we present an inexpensive and compact device that quantifies hazardous methanol accurately in hand sanitizers by headspace analysis. It comprises a separation column (van den Broek et al., 2020c) of Tenax TA particles and a chemoresistive gas sensor of Pd-doped SnO_2_ nanoparticles (van den Broek et al., 2019) integrated into a smartphone-assisted analyzer with validated performance for alcoholic drinks (Abegg et al., 2020). Here, we applied it to seven pure and methanol-spiked (0.01–90 vol%) commercial hand sanitizers (total 76 samples) with various compositions (Table 1) to assess its resistance to challenging 2-propanol, glycerol, various odorants, and gel-like viscosity. Results were compared to established gas chromatography as recommended by FDA (U.S. Food and Drug Administration, 2020b).

## Results and discussion

### Analytical strategy

**Figure 1:**
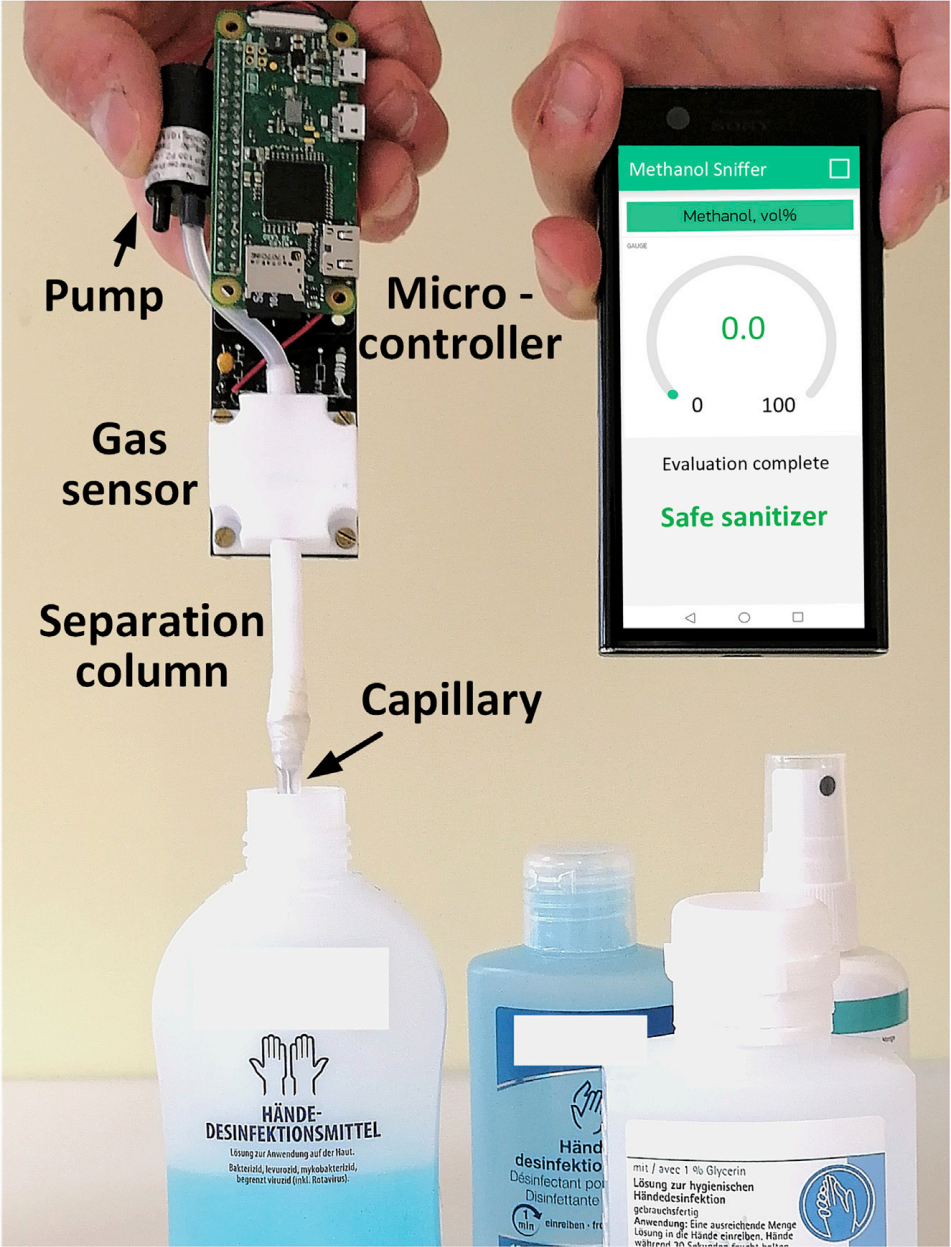
Handheld methanol detector for screening hand sanitizers Key components are the capillary for vapor sampling, separation column, gas sensor (sealed by chamber), pump and microcontroller. Data are communicated wirelessly to a smartphone and an exemplary user interface is shown.

The handheld device is shown in Figure 1. For hand sanitizer analysis, headspace vapor is extracted for 10 s through a sampling capillary with a vane pump. When transported through the separation column (i.e. packed bed of non-polar Tenax TA polymer particles), the analytes are separated by sorption (similar to gas chromatography) on the Tenax TA's available surface area (van den Broek et al., 2019) of 35 m^2^ g^−1^. Specifically, larger alcohols (e.g. ethanol, 2-propanol), the main constituents of hand sanitizers (Table 1), are retained longer than methanol due to stronger van der Waals adsorption forces (Maier and Fieber, 1988) rendering the device selective. This represents a key challenge for conventional chemical sensors that can hardly distinguish these molecules (Guo et al., 2011) due to their chemical similarity (i.e. hydroxyl group).

A chemoresistive microgas sensor downstream of the separation column detects and quantifies the methanol content. It is based on a porous film, self-assembled by flame-aerosol deposition of SnO_2_ nanoparticles (grain size 16 nm (Abegg et al., 2020)) containing lattice-incorporated and surface-loaded Pd (Pineau et al., 2020) that feature high sensitivity to various volatile organics (e.g. down to 3 ppb formaldehyde at 90% relative humidity (Güntner et al., 2016)) but cannot distinguish methanol from other alcohols without the separation column (van den Broek et al., 2019). Methanol is adsorbed on these nanoparticles (Ouyang et al., 2000) and converted by chemical reaction with oxygen- and hydroxyl-related species (Cheong and Lee, 2006). The associated release of electrons into the n-type semiconducting SnO_2_ results in a measurable signal (i.e. film resistance change) (Ogawa et al., 1982) that is proportional to the methanol concentration. All other parts of the device in contact with analytes (e.g. tubing, sensor housing, etc.) are made of inert Teflon to minimize adsorption and contamination. After flushing the column and sensor with ambient air to remove residual adsorbate, it can be reused after 15 min and provided stable results during more than three months of repeated testing (Abegg et al., 2020).

### Selective methanol detection over other alcohols

**Figure 2:**
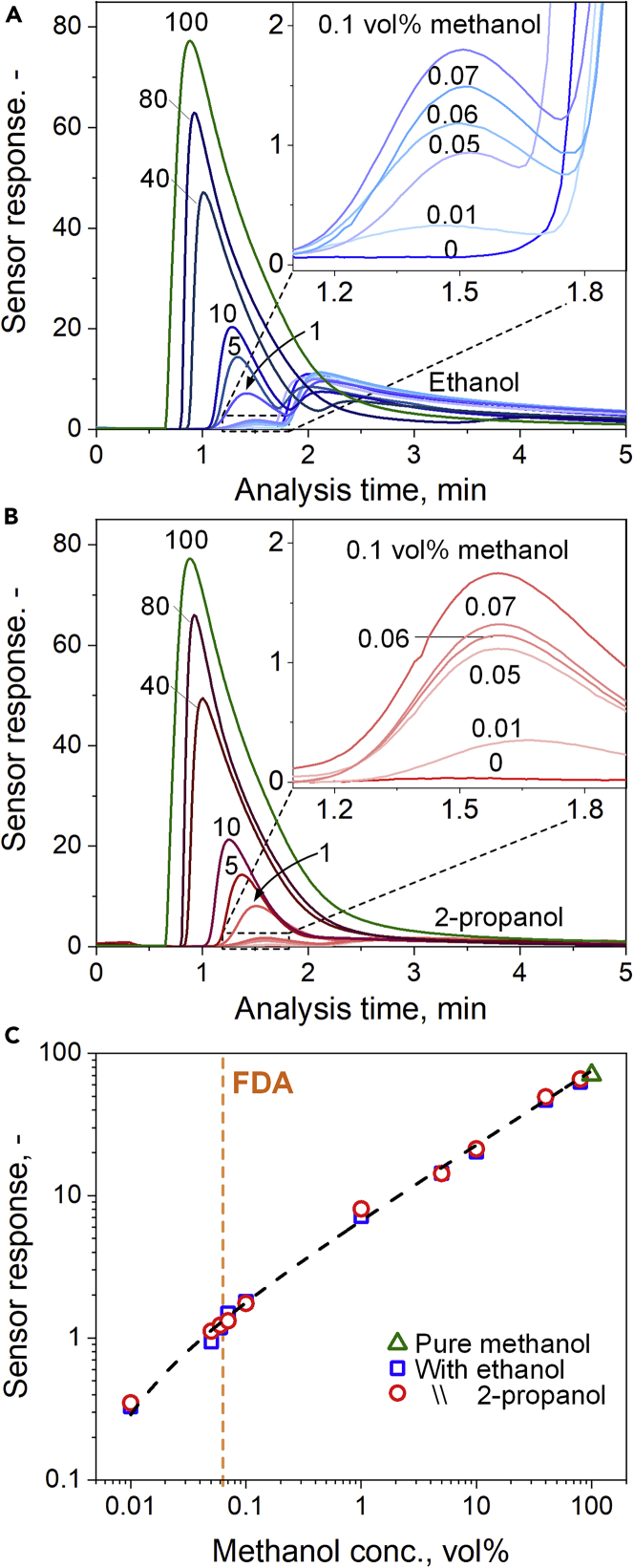
Methanol detection in ethanol and 2-propanol mixtures (A and B) Sensor response to 0–100 vol% methanol in ethanol (A) or 2-propanol (B). Insets magnify 0–0.1 vol% methanol. (C) Sensor response peak values for pure methanol (triangle) and with ethanol (squares) or 2-propanol (circles). Indicated is also the FDA recommended limit (i.e. 0.063 vol%, vertical dashed line) and best fit (black dashed line).

Figure 2A shows the sensor response curves for 0–100 vol% methanol in ethanol. Methanol passes through the separation column first with retention times (t_R_) between 1.5 and 0.8 min for 0.01–100 vol%, respectively, in agreement with literature (i.e. 1.25 min for 10 vol% methanol in 80 vol% ethanol and water (Abegg et al., 2020)). Note that shorter retention times with increasing methanol levels are due to an overloading of the column, as with gas chromatography (Yabumoto et al., 1980), but this does not affect methanol quantification, as shown below. Most importantly, ethanol elutes later (t_R_ = 2 min for pure ethanol, Figure S1) without interfering the methanol measurement. Similarly, 2-propanol (Figure 2B) passes the separation column even later (t_R_ = 2.8 min for pure 2-propanol, Figure S1) with rather small response. As a result, methanol is detected selectively over these alcohols overcoming a major bottleneck in chemical sensing.

Another challenge is the quantification of methanol over a large concentration range: at least from 0.063 vol% (U.S. Food and Drug Administration, 2020b) (FDA limit) to 81 vol% (max. content found in adulterated sanitizers (U.S. Food and Drug Administration, 2020a)). This is met by the device that detects methanol over four orders of magnitude (0.01–100 vol%, Figure 2C) with almost identical responses (average deviation of 4%, R^2^ = 0.99) in ethanol (squares) and 2-propanol (circles), highlighting again its excellent selectivity. Remarkably, even lowest 0.01 vol% (Insets, Figures 2A and 2B) are detected with high signal-to-noise (> 300) within 2 min at very high alcohol background (i.e. > 99 vol%). The recognition of such low methanol concentrations is superior to state-the-art sensors (Table 2) featuring higher detection limits in liquids, for instance, electrochemical cells (Ou et al., 2019) (0.15 vol%) or fluorescent sensors (Huang et al., 2018) (4 vol%). Also close to the FDA limit, methanol concentrations are distinguished clearly, as demonstrated for 0.05, 0.06, and 0.07 vol% (Insets, Figures 2A and 2B). Please note that the t_R_ at such low methanol concentrations are slightly higher (e.g. 1.6 vs. 1.5 min at 0.06 vol%) in 2-propanol than ethanol, probably due to competitive adsorption (Comes et al., 1993) on the Tenax TA and the higher vapor pressure of ethanol.

### Hand sanitizers

**Figure 3:**
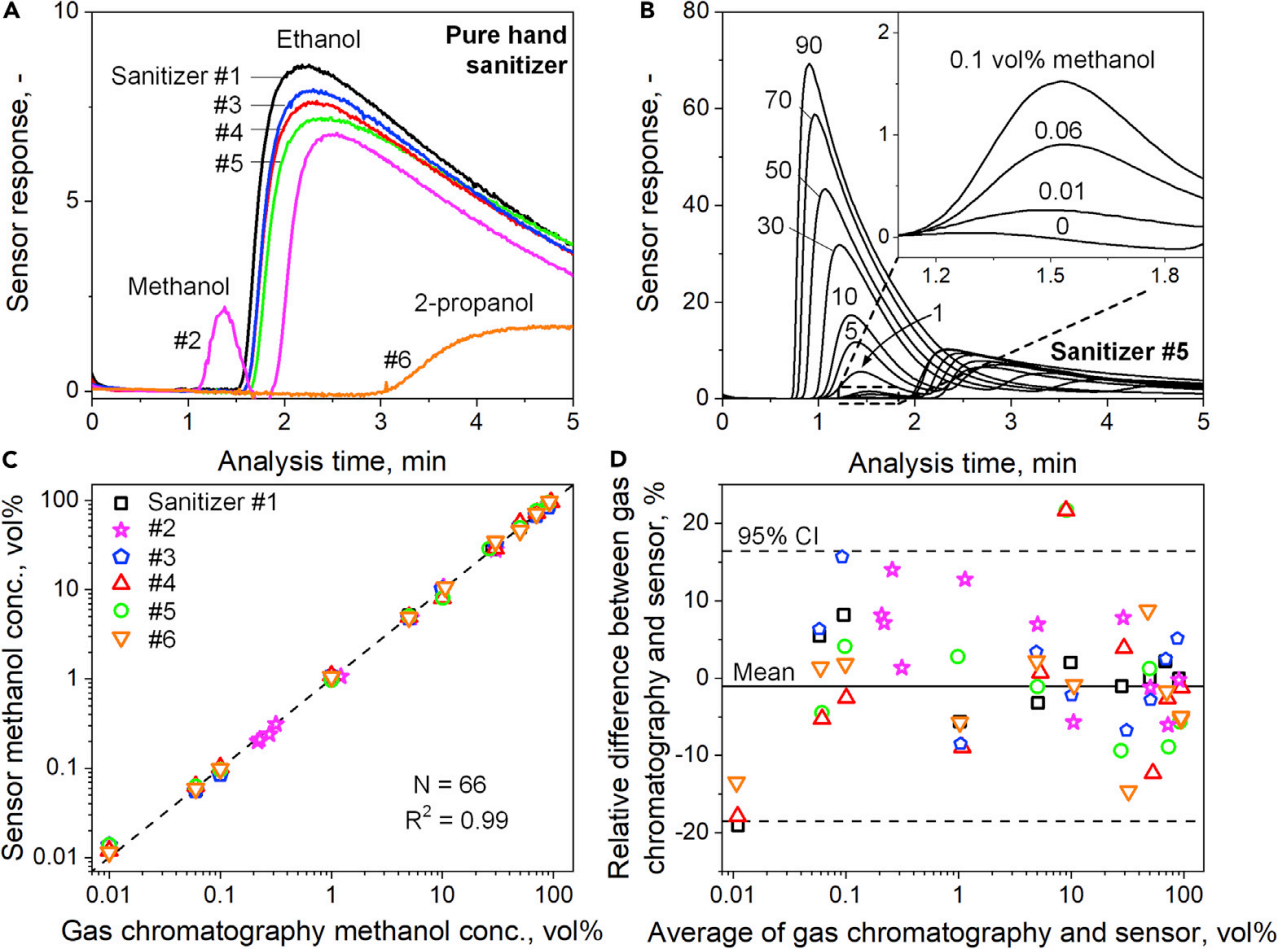
Commercial hand sanitizers evaluated by sensor and gas chromatography (A) Sensor response to the commercial hand sanitizers with different compositions (Table 1). Associated peaks for methanol, ethanol, and 2-propanol are indicated. (B) Response to 0–90 vol% methanol-spiked samples of sanitizer #5 that contains 81 vol% ethanol, water, glycerol, panthenol, cyclopentasiloxane, cyclohexasiloxane, isotrideceth-8, 2-propanol, and didecyldimethylammoniumchloride (Table 1). Inset shows magnification of 0–0.1 vol% methanol content. (C) Scatterplot (66 samples) indicating the methanol content in pure and spiked hand sanitizers, as measured by sensor and gas chromatography. (D) Corresponding Bland-Altman analysis (Martin Bland and Altman, 1986) indicating the relative difference of the measured methanol concentrations vs. the average concentration of both instruments. Mean and limits of agreement (95% confidence intervals, CIs) are provided as solid and dashed lines, respectively.

Hand sanitizers are typically more complex mixtures containing also humectants, odorants, denaturants, and colorants. Thus, the device was evaluated (Figure 3A) on six commercially available hand sanitizers with different compositions (Table 1), as characterized also by gas chromatography (Figure S2). Sanitizers #1–5 are ethanol-based, as correctly recognized by the device. On the other hand, hand sanitizer #6 contains mainly 2- (49 vol%) and 1-propanol (32 vol%) with both compounds being identified by the sensor (Figure S3). It should be noted that the FDA considers 1-propanol toxic (U.S. Food and Drug Administration, 2020b) and has limited its content also to 0.1 vol% while it is recommended as active substance in biocidal products in the E.U (European Chemical Agency, 2020). No other distinct peaks are detected, so other compounds that elute earlier than methanol (e.g. formaldehyde (van den Broek et al., 2020b)) do not interfere with the measurement.

Only sample #2 contained detectable amounts of methanol, as recognized by the device with a response of 2.2 at (t_R_) 1.4 min and confirmed by gas chromatography (0.19 vol%, Figure S2). This hand sanitizer is based on fruit-derived distillates where methanol is formed naturally during fermentation (from pectin degradation (Bindler et al., 1988)). Please note that its methanol content, however, is below the E.U. limit (i.e. 0.9 vol% at that ethanol content (European Parliament and Council, 2019)) for fruit distillates.

Next, these hand sanitizers were spiked with 0.01–90 vol% methanol (total 66 samples) to simulate the entire range of typical contamination/adulteration. Figure 3B shows the sensor response exemplarily for sample #5 that contains 81 vol% ethanol (Table 1) but also glycerol, panthenol, cyclopentasiloxane, cyclohexasiloxane, isotrideceth-8, 2-propanol, and didecyldimethylammoniumchloride (please see Figure S4 for sample #3). Remarkably, these compounds do not interfere the measurement. In fact, methanol elutes at comparable t_R_ to the binary mixtures with ethanol (Figure 2A) and is quantified with similar response (1.5 vs. 1.7 for 0.1 vol% methanol). We confirmed this also through experiments with pure substances (Figure S1), where other compounds were detected only after 2 min being higher than the methanol t_R_ for lowest 0.01 vol% (i.e. 1.5 min).

Figure 3C shows the methanol concentrations of pure and spiked hand sanitizers, as measured by our detector and “gold standard” gas chromatography. The detector quantifies methanol accurately over four orders of magnitude with high R^2^ of 0.99. The error is fairly small (95% confidence interval: −18.5 to 16.4%, dashed lines in Figure 3D) and stays rather constant over the entire measurement range, as revealed by Bland-Altman analysis (Martin Bland and Altman, 1986). In other words, methanol concentrations at the FDA limit (0.063 vol%) will be determined between 0.051 and 0.073 vol%, which should be sufficiently accurate for screening hand sanitizers. Consequently, methanol is detected reliably in the commercial hand sanitizers #1-6 despite their different compositions (Table 1). Also, colorants (e.g. #6 contains patent blue V) do not interfere the measurement (Figure 3C, inverse triangles), that may be quite problematic for colorimetric tests (e.g. Alert for Methanol).

**Figure 4:**
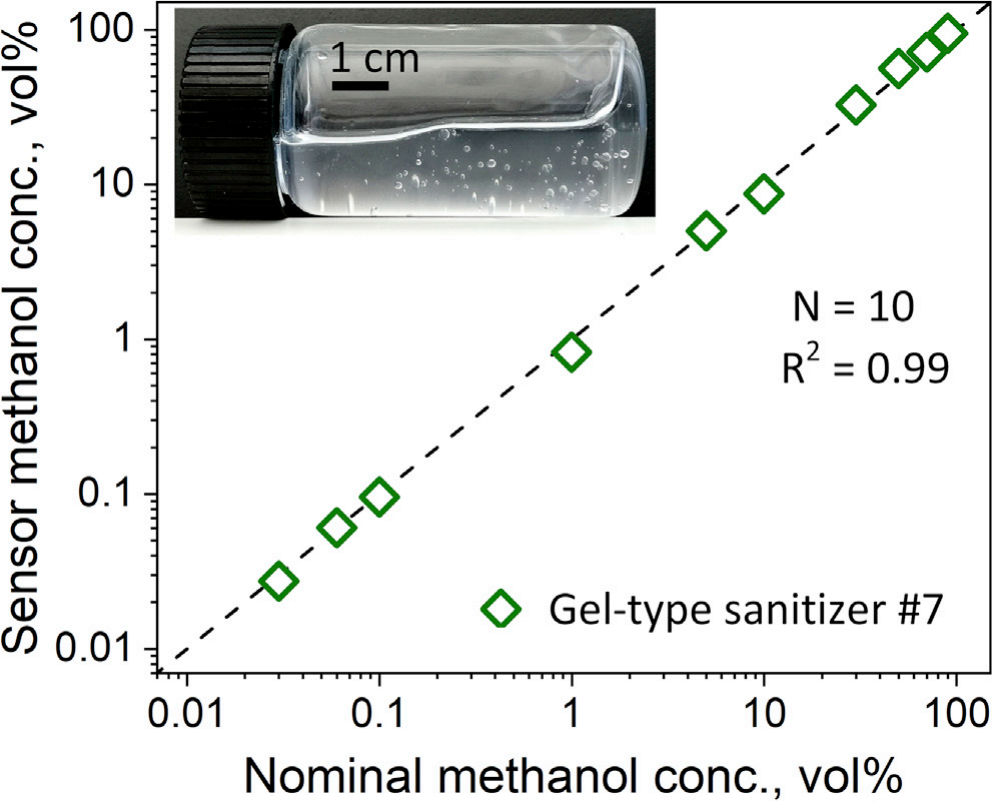
Gel-like hand sanitizer #7 Methanol concentration measured by the sensor in gel-like hand sanitizer #7 (methanol-spiked). Note that direct analysis by gas chromatography was not feasible due to the sanitizer's high viscosity. Inset shows the sample.

Finally, we tested also the gel-like hand sanitizer #7 (Figure 4) to assess viscosity effects. Most importantly, the spiked methanol concentrations were recognized well with high (0.99) R^2^, consistent to the less viscous samples #1 - 6 (Figure 3C). This highlights the robustness of present headspace analysis even for highly viscous samples, where commercial colorimetric assays might fail, as indicator solutions do not mix well with such fluids.

We anticipate this device to be helpful to police, customs, distributors, and consumers to check product safety. It is compact (2 × 4 × 12 cm^3^, Figure 1), weighs only 94 g and offers low power consumption (ca. 1.1 W during analysis) enabling battery-driven operation (Abegg et al., 2020). A first, rough cost estimation based on its key commercially available components (Table S1) suggests a unit price of 137 USD. Note that the component costs were obtained from suppliers when ordered at small numbers (<10), that should drop significantly at higher quantities making the device affordable for a broad population even in low-income countries. The operation and data display are user-friendly by providing wireless communication by Wi-Fi or Bluetooth, functioning even if no external network is available. When combined with a breath sampler, this device is even applicable for medical screening of methanol poisoning by noninvasive (Güntner et al., 2019) breath analysis (van den Broek et al., 2020a), as established for ethanol by law enforcement.

### Conclusions

We presented a handheld and readily applicable detector for distributed and on-site screening of sanitizers for toxic methanol. It quantifies methanol within two minutes selectively over four orders of magnitude (0.01–100 vol%) and meets even newest national guidelines (e.g. FDA), as validated by gas chromatography. Typical hand sanitizer constituents and gel-like viscosity do not interfere the measurement while other potential contaminants (e.g. 1-propanol) are recognized as well. The device operation and data analysis is user-friendly, providing results on smartphones, where further communication to data clouds for remote analysis is possible. The device contains mostly commercially available components, thus can be produced at low cost and large numbers. It addresses an urgent need during the COVID-19 health crisis where widespread access to safe sanitizers is crucial to mitigate disease propagation.

### Limitations of the study

We had investigated the detection of methanol in pure and artificially spiked hand sanitizers of various compositions under rather controlled laboratory conditions. Therefore, field tests are required to assess further potential interferences. For instance, temperature and relative humidity are known to affect the separation performance of the column and the methanol sensitivity of the sensor, as had been investigated between 22 and 40 °C and 10–90%, respectively (van den Broek et al., 2019). However, these can be corrected with colocated temperature and humidity sensors (Güntner et al., 2018).

### Resource availability

#### Lead contact

Further information and requests for resources and materials should be directed to and will be fulfilled by the lead contact, Andreas T. Güntner (Andreas.guentner@ptl.mavt.ethz.ch).

#### Materials availability

This study did not yield new unique reagents.

#### Data and code availability

This study produced a device program code that is provided in the Supplemental information.

## Methods

All methods can be found in the accompanying Transparent methods supplemental file.

## Supplemental information

Document S1. Transparent methods, Figures S1–S5, and Table S1

## Footnotes

Supplemental information can be found online at https://doi.org/10.1016/j.isci.2021.102050.
