# Supplementary material for: Detecting methanol in hand sanitizers
Source: iScience. 2021 Jan 12;24(2):102050. doi: 10.1016/j.isci.2021.102050 (PMC7840468; doi:10.1016/j.isci.2021.102050)
Supplement: Document S1. Transparent methods, Figures S1–S5, and Table S1 [file mmc1.pdf]

iScience, Volume *24*

Supplemental Information

Detecting methanol in hand sanitizers

Andreas T. Güntner, Leandro Magro, Jan van den Broek, and Sotiris E. Pratsinis

TRANSPARENT METHODS

Device design

The handheld detector is shown in Figure 1, a component cost estimate in Table S1 and its design elaborated elsewhere (Abegg et al., 2020). In brief, vapor from the headspace of liquid samples was extracted with a capillary (Sterican, B. Braun, Germany) fixed to a Teflon tube (4 mm inner diameter). This tube contained the sorption material, 150 mg Tenax TA powder (60–80 mesh, ~35 m^2 g^{-1}, poly(2,6-diphenyl-p-phenylene oxide), Sigma Aldrich, Switzerland) (van den Broek et al., 2019), that was fixed as packed bed with tension springs and silanized glass wool plugs to avoid voids. Note that such separation columns could be miniaturized even further by microfabrication and their loading can be varied flexibly to adjust analyte separation for other analytes (e.g. formaldehyde (van den Broek et al., 2020)). A vane pump (135 FZ 3 V, Schwarz Precision, Germany) provided the flow for sampling and flushing to recover the separation column.

The gas sensor consists of Pd-doped SnO_2 nanoparticles made by flame spray pyrolysis and directly deposited onto micromachined sensor substrate (Güntner et al., 2016) (1.9×1.7 mm^2, MSGS 5000i, Microsens SA, Switzerland) featuring interdigitated electrodes and a heater on a free-standing membrane. This sensor was mounted onto a leadless chip carrier (LCC, Chelsea Technology Inc., U.S.A.) with high temperature carbon paste (Ted Pella Inc., U.S.A.) and electrically connected through aluminum wires (30 μm in diameter) by bonding (F&K Delvotec, Germany). After placing it on a socket (E-Tec, Switzerland) that was soldered to a printed circuit board (PCB), the sensor was sealed (gas-tight) by an inert Teflon chamber with its design disclosed elsewhere (Abegg et al., 2020). A microcontroller (Raspberry pi Zero W, U.S.A.) provided the required heating power to operate the sensor at 350 °C (van den Broek et al., 2019), monitored its resistance and communicated data wirelessly to a smartphone by Bluetooth or Wi-Fi. The device program code for

communication between the device and the smartphone is provided below. The smartphone prototype app was made with a free mobile app constructor (Version 2.27.19, Blynk Inc., U.S.A.). Blynk offers a streamlined interface with a library of user interface components (e.g. buttons) that send and receive data from the device. These components can be directly arranged and configured via the Blynk app, allowing simple extension of the app with additional functionalities.

Sample preparation

The applied substances were methanol (> 99.9%, Sigma-Aldrich, Germany), ethanol (> 99.8%, Fisher Chemical, Switzerland), 1-propanol (> 99%, Merck, Germany), 2-propanol (> 99.5%, Sigma Aldrich, Germany), butanone (> 99%, VWR International, France) and Milli-Q water (Milli-Q Synthesis A10, Merck, Germany). Also seven commercial hand sanitizers were tested with their identifiers, producers and compositions, as available, listed in Table 1. Binary, ternary (for calibration) mixtures and methanol-spiked hand sanitizers were obtained by admixing the desired amounts of methanol with high precision pipettes. Each sample was 5 mL prepared in 20 mL glass vials (Vial SCR 20ML, VWR, Germany) leaving sufficient headspace for vapor analysis. The vials were sealed immediately after preparation with caps (polypropylene screw cap with hole 24 mm, Supelco, U.S.A.) containing a septum (Teflon faced silicone septa 22 mm, Supelco, U.S.A.), unless otherwise stated.

Headspace analysis

Right before each sensor measurement, the prepared vials were rigorously shaken (at least 30 s) to afford phase equilibrium in the vial (Abegg et al., 2020). Next, the capillary of the detector was inserted through the vial septum together with a second capillary for pressure balance. Note that sampling can be done also from the open container (Figure 1), though this is less accurate (Figure S5) due to higher dilution with surrounding air. Sample was extracted always for 10 s at a sampling rate of 25 mL min^{-1} drawn by the vane pump. Afterwards the

capillary was removed from the vial and ambient air was drawn continuously to transport the sample through the separation column and to the sensor. By flushing with ambient air at 65 mL min^{-1}, residual adsorbate was removed from the separation column to facilitate fast detector reusability. After recovery, the flow rate was set to zero to reduce the amount of noise due to ambient air interferants (Abegg et al., 2020).

The dimensionless sensor response (S) was defined as:

$$(1)\quad S = \frac{R_b}{R_s} - 1$$

with R_b and R_s being the sensor (i.e. Pd-doped SnO_2 film) resistances at baseline (stabilized in room air) and under sample exposure, respectively. The t_R of an analyte was defined as the time required to reach the response peak, similar to gas chromatography (Geankoplis, 2003). The methanol concentration in pure and spiked hand sanitizers were quantified by comparing the peak response to five-point calibration curves from methanol-ethanol-water mixtures (giving similar methanol responses to mixtures with 2-propanol instead of ethanol, Figure 2c) in the expected concentration range, as elaborated elsewhere (Abegg et al., 2020).

The methanol content of pure and spiked hand sanitizers #1-6 was determined also by gas chromatography for comparison. Note that gel-type hand sanitizer #7 was not analyzed due to its high viscosity. Measurements were performed on a Varian 3800 (Agilent, U.S.A.) with a column (Zebron ZB-624, Brechbühler AG, Switzerland) and flame ionization detector operated at 45 and 220 °C, respectively. The sampling volume and pressure were 0.5 μL and 4 psi, respectively and the injector was applied at 210 °C with split ratio 20. Methanol concentrations were obtained by comparing the area under curve of the methanol signal to calibration curves, as evaluated with the software Varian Star Chromatography Workstation (Agilent, U.S.A.). The calibration was done with the above-mentioned standards by mixing the desired amounts with precision graduated and volumetric pipettes (Hirschmann, Germany) in a 100 mL volumetric flask and analyzing the peak response area (McNair et al., 2019).

Device program code (Related to Figure 1)

```
#!/usr/bin/env python3
# -*- coding: utf-8 -*-

import time
from gpiozero import MCP3208  # Analog to digital converter, v1.5.0
import pigpio  # Raspberry PI GPIO pin control, v1.38
p = pigpio.pi()

from simple_pid import PID  # PID module, v0.2.4

import blynklib  # Blynk smartphone app communication, v0.2.6
blynk = blynklib.Blynk('<code>')  # Unique authenitication code from the Blynk app

pin_heater = 12  # Power supply pin for sensor heater
pin_pump = 13  # Power supply pin for pump
freq = 100000  # Frequency of pulsed width modulation (Hz)
duty_heater = 0.4  # Initial duty cycles for sensor heater (0-1)
duty_pump = 0.7  # Initial duty cycles for pump (0-1)

power_setpoint = 85  # Sensor heater power setpoint (mW)
sample_time = 1  # Sampling period (s)

R_sensor_ref = 999000  # Reference resistance for sensor voltage divider (Ohm)
R_heater_ref = 56  # Reference resistance for heater voltage divider (Ohm)
V_ref = 3.280  # Voltage at gpio for voltage divider
gain = 1.69  # Operational amplifier gain

adc_sensor = MCP3208(channel=0, device=0)  # Initialize sensor ADC
adc_heater = MCP3208(channel=2, device=0)  # Initialize heater ADC

pid = PID(1, 0.1, 0.05, setpoint=power_setpoint) # Initialize PID controller

# Called from button in Blynk app connected to virtual pin V1
@blynk.handle_event('write V1')
def pump_button(pin, value):
        if value[0] == '1': p.hardware_PWM(pin_pump, freq, int(duty_pump * 1E6))
        else: p.hardware_PWM(pin_pump, freq, 0)

# Measurement loop
while True:
        # Calculate sensor heater power consumption (PC)
        V_heater = V_ref * adc_heater.value
        V_applied_heater = duty_heater * V_ref * gain
        PC = V_heater * (V_applied_heater - V_heater) / R_heater_ref * 1000

        # Power Control +/-1mW ~ +/-0.5%
        duty_heater += 0.005 * pid(PC)
        p.hardware_PWM(pin_heater, freq, int(duty_heater * 1E6))

        # Calculate sensor resistance
        V_sensor = V_ref * adc_sensor.value
        R_sensor = V_sensor * R_sensor_ref / (V_ref - V_sensor)

        blynk.run()  # Calls method pump_button if pump was started from the app
        # Sends sensor resistance to Blynk component connected to virtual pin V0
        blynk.virtual_write(0, round(R_sensor))

        time.sleep(sample_time) # Wait for next measurement
```

SUPPLEMENTAL DATA ITEMS

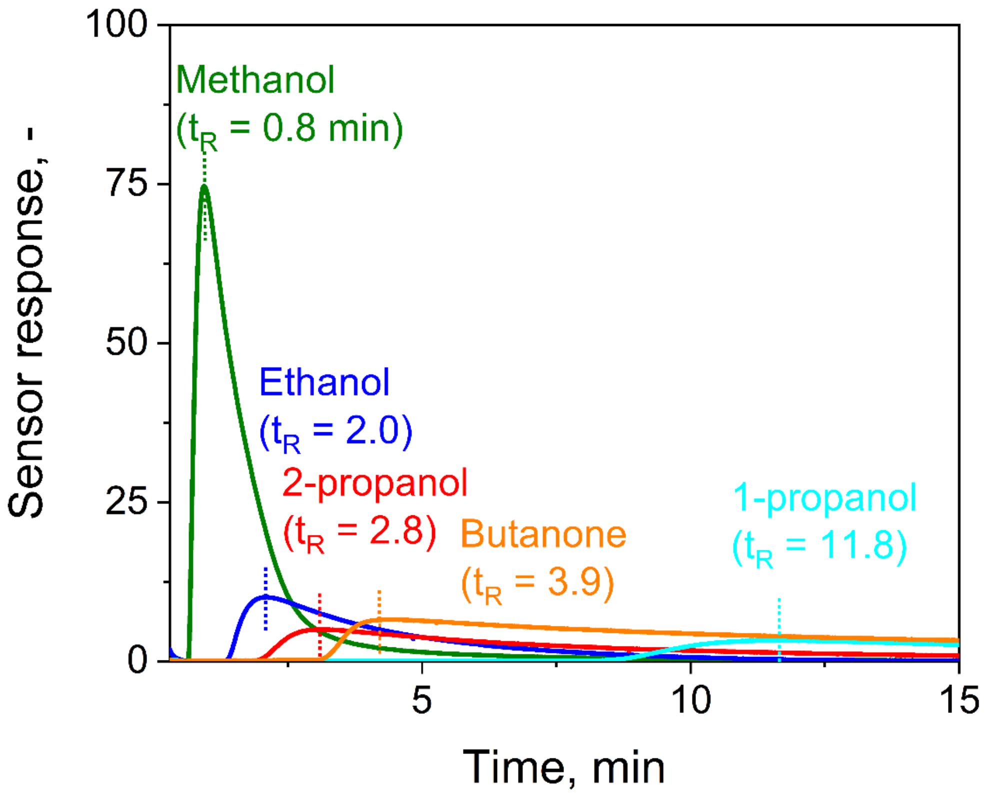

Figure S1. Sensor response to sanitizer-related pure substances (Related to Figure 2)

Indicated with dashed lines are the individual t_R with values in brackets.

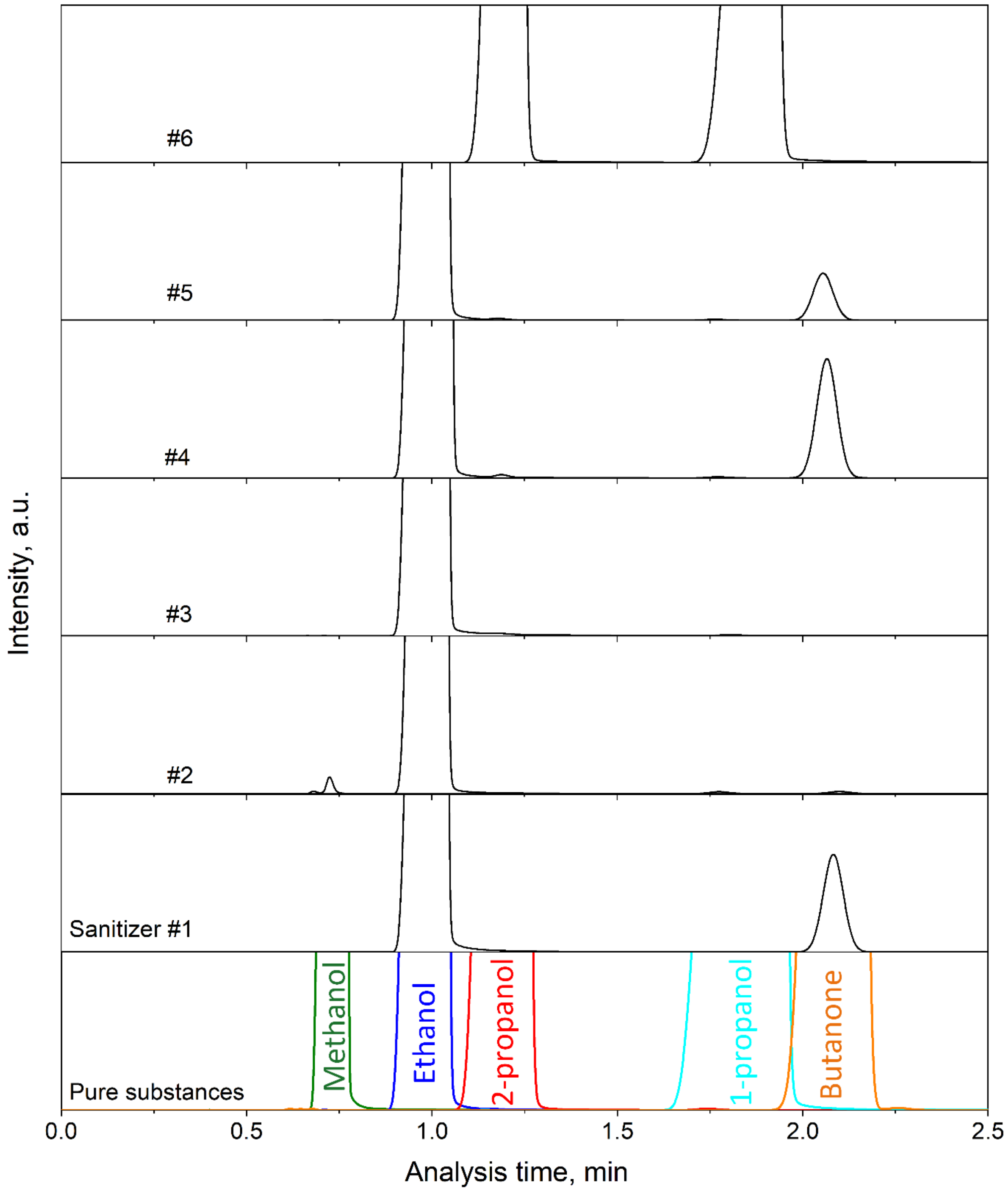

Figure S2. Gas chromatograms of commercial hand sanitizers (Related to Figure 3)

Hand sanitizers #1-6 (Table 1) and pure substances as reference (bottom graph).

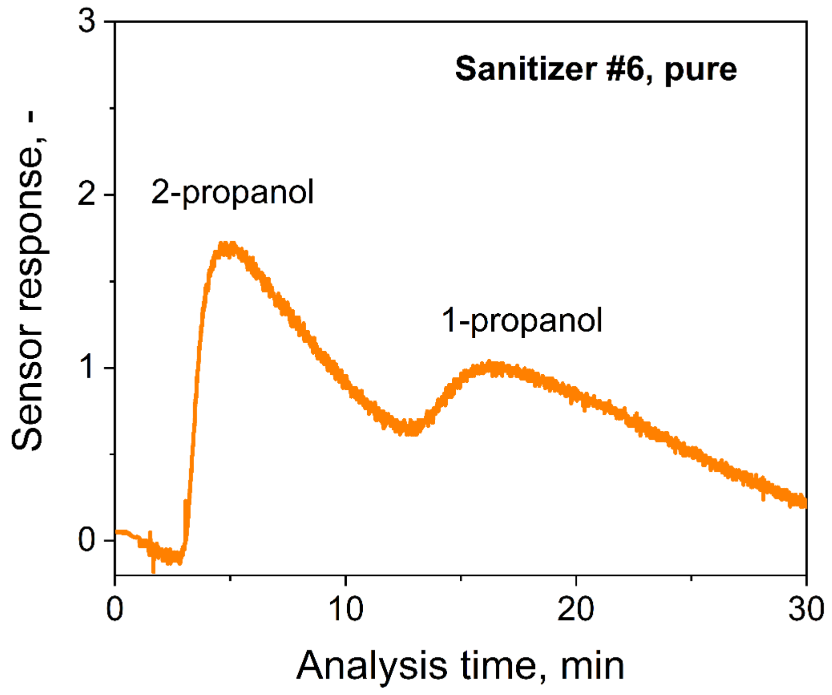

Figure S3. Sensor response to pure sanitizer #6 (Related to Figure 3)

Peaks of 2- and 1-propanol are labelled.

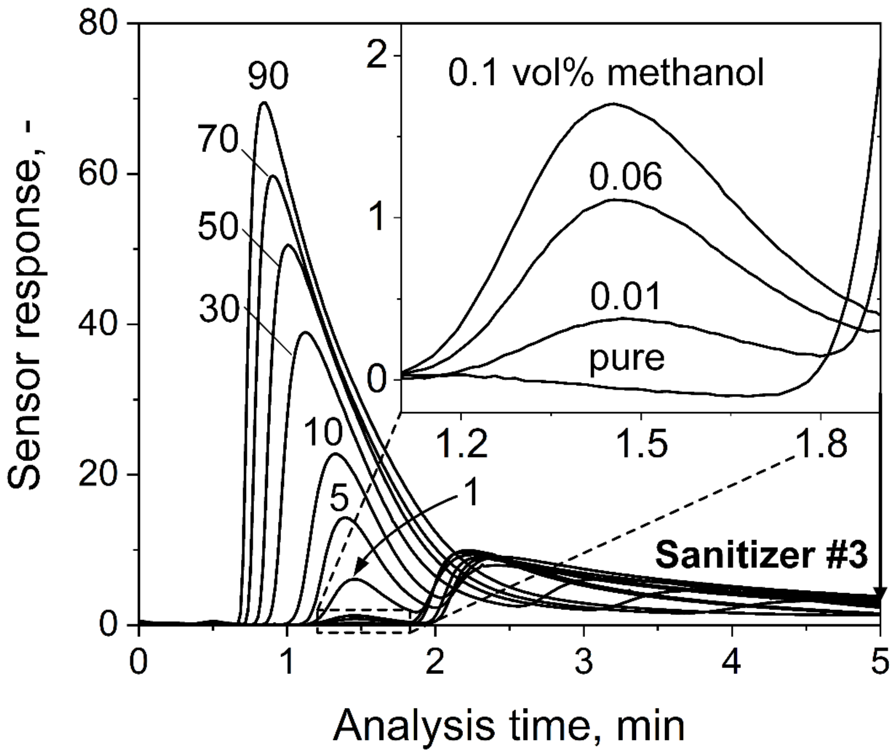

Figure S4. Sensor response to hand sanitizer #3 (Related to Figure 3)

Sensor response to 0 – 90 vol% methanol-spiked sanitizer #3 that contains 82 vol% ethanol. Inset shows magnification of 0 – 0.1 vol% methanol content.

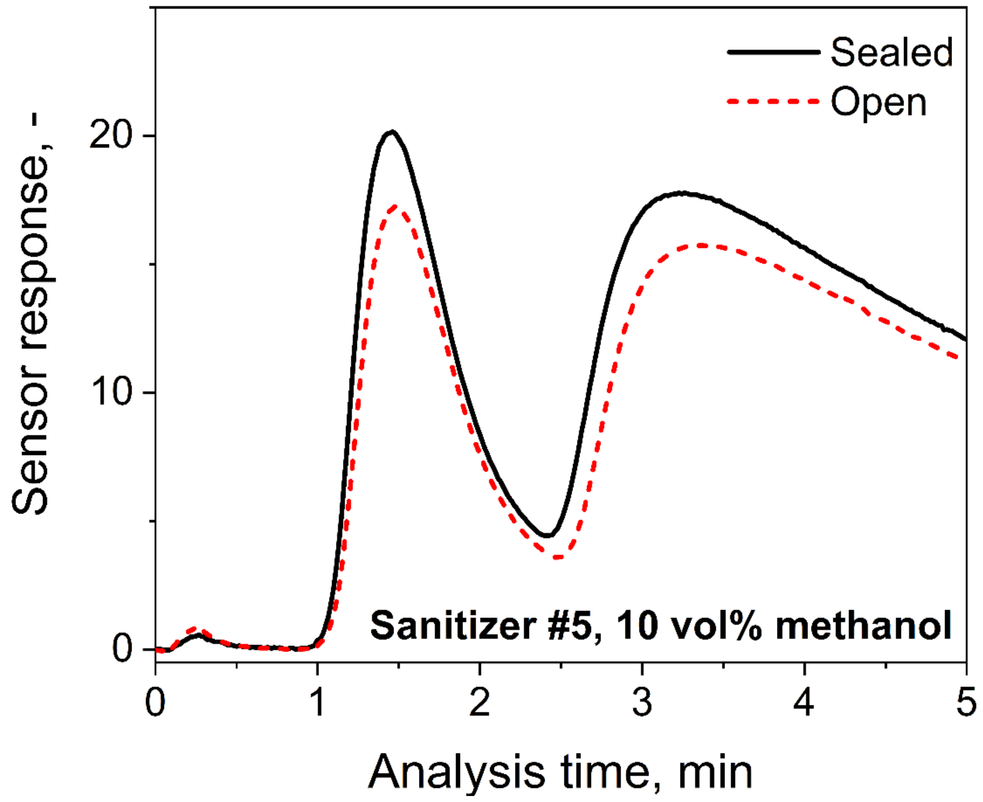

Figure S5. Sampling of hand sanitizer #5 with sealed and open vial (Related to Figure 3) Detector sampling with sealed (black solid line, from Figure 3b) and open (red dashed line) vial of sanitizer #5 spiked with 10 vol% methanol. Before, samples were shaken for at least 30 s. For the *sealed* measurement, the septum remained on the vial and was penetrated by the capillary (see Transparent Methods above). In case of *open*, the septum was removed for instantaneous sample extraction. The methanol peak response difference between sealed and open measurement was 14%.

Table S1. Price estimate of the methanol detector components (Related to Figure 1)

Costs of the key device components ordered at small quantities (<10 pcs.). Note that sensor is homemade, so its price was estimated from a comparable (i.e. chemoresistive, metal oxide-based) commercial sensor.

Component	Type	Price (USD)	Supplier
Microcontroller	Raspberry Pi Zero W	10	www.raspberrypi.org
Separation column	150 mg Tenax® TA	4.1	www.sigmaaldrich.com
Sensor	BME680, Bosch	11.8	www.mouser.com
Pump	135 FZ 3V, Schwarz Precision	100	www.schwarzer.com
PCB	Custom-design	11.3	www.pcbway.com
**Total**		**137.2**	

SUPPLEMENTAL REFERENCES

Geankoplis, C.J. (2003). Transport processes and separation process principles:(includes unit operations) (Prentice Hall Professional Technical Reference).

McNair, H.M., Miller, J.M., and Snow, N.H. (2019). Basic gas chromatography (John Wiley & Sons).
